# Efficicent (*R*)-Phenylethanol Production with Enantioselectivity-Alerted (*S*)-Carbonyl Reductase II and NADPH Regeneration

**DOI:** 10.1371/journal.pone.0083586

**Published:** 2013-12-17

**Authors:** Rongzhen Zhang, Botao Zhang, Yan Xu, Yaohui Li, Ming Li, Hongbo Liang, Rong Xiao

**Affiliations:** 1 Key Laboratory of Industrial Biotechnology of Ministry of Education & School of Biotechnology, Jiangnan University, Wuxi, P. R. China; 2 National Key Laboratory for Food Science, Jiangnan University, Wuxi, P. R. China; 3 Tianjin Institute of Industrial Biotechnology, The Chinese Academy of Sciences, Tianjin, P. R. China; 4 Center for Advanced Biotechnology and Medicine, Rutgers University, Piscataway, New Jersey, United States of America; Russian Academy of Sciences, Institute for Biological Instrumentation, Russian Federation

## Abstract

The NADPH-dependent (*S*)-carbonyl reductaseII from *Candida parapsilosis* catalyzes acetophenone to chiral phenylethanol in a very low yield of 3.2%. Site-directed mutagenesis was used to design two mutants Ala220Asp and Glu228Ser, inside or adjacent to the substrate-binding pocket. Both mutations caused a significant enantioselectivity shift toward (*R*)-phenylethanol in the reduction of acetophenone. The variant E228S produced (*R*)-phenylethanol with an optical purity above 99%, in 80.2% yield. The E228S mutation resulted in a 4.6-fold decrease in the *K*_*M*_ value, but nearly 5-fold and 21-fold increases in the *k*_cat_ and *k*_cat_/*K*_M_ values with respect to the wild type. For NADPH regeneration, *Bacillus* sp. YX-1 glucose dehydrogenase was introduced into the (*R*)-phenylethanol pathway. A coexpression system containing E228S and glucose dehydrogenase was constructed. The system was optimized by altering the coding gene order on the plasmid and using the Shine–Dalgarno sequence and the aligned spacing sequence as a linker between them. The presence of glucose dehydrogenase increased the NADPH concentration slightly and decreased NADP^+^ pool 2- to 4-fold; the NADPH/NADP^+^ ratio was improved 2- to 5-fold. The recombinant *Escherichia coli*/pET-MS-SD-AS-G, with E228S located upstream and glucose dehydrogenase downstream, showed excellent performance, giving (*R*)-phenylethanol of an optical purity of 99.5 % in 92.2% yield in 12 h in the absence of an external cofactor. When 0.06 mM NADP^+^ was added at the beginning of the reaction, the reaction duration was reduced to 1 h. Optimization of the coexpression system stimulated an over 30-fold increase in the yield of (*R*)-phenylethanol, and simultaneously reduced the reaction time 48-fold compared with the wild-type enzyme. This report describes possible mechanisms for alteration of the enantiopreferences of carbonyl reductases by site mutation, and cofactor rebalancing pathways for efficient chiral alcohols production.

## Introduction

Optically active alcohols are very useful chiral blocks in the special chemical and pharmaceutical industries [1–3]. The cofactor-dependent asymmetric reduction of ketones catalyzed by alcohol dehydrogenases, is a valuable method for the synthesis of optically active alcohols [4,5]. For example, a short-chain dehydrogenase/reductase (SDR), NADPH-dependent (*S*)-carbonyl reductaseII (SCRII) from *Candida parapsilosis* CCTCC M203011 catalyzes the biotransformation of (*S*)-1-phenyl-1,2-ethanediol (PED) from β-hydroxyacetophenone [6,7]. However, many alcohol dehydrogenases have the limitations of a narrow substrate specificity and the need for expensive nicotinamide cofactors in stoichiometric amounts for applications [8,9]. It is essential to tailor an enzyme or redesign its new functions [10], and then carry out biocatalytic reduction processes preferably in the absence of an external cofactor, using substrate-coupled and enzyme-coupled techniques [9,11,12].

Recently, the structural studies of SDR enzymes have helped us to understand the principles underlying their substrate specificities, enabling broadening of their substrate spectrums or alteration of their substrate enantioselectivities by site-mutations [5,13,14]. The substrate-binding loop located at distal to the αF is always an active region for mutation for tailoring SDR enzymes and changing their enantiopreferences [14]. In recent years, many groups have redesigned enzyme functions and improved enzyme properties such as stereoselectivity and activity, and altered their catalytic activity alteration by site-mutagenesis [10,15]. Flanagan and Masuda et al. reported the formation of a novel metabolite of dextromethorphan and product enantioselectivity alteration by Phe120Ala in cytochrome P450 2D6 [16,17]. 

Although many catalysts including the microbial cell systems and carbonyl reductases have been reported to have improved functions after rational design, there are suffer several problems with regards to achieving effective enzymatic reduction. The most striking problems involve supply of the coenzyme [18,19]. Many oxidoreductases require an expensive cofactor, NAD(H) or NADP(H) to act as stoichiometric agents in biotransformation reactions and undergo chemical reactions with substrates. So, efficient regeneration or reuse of the cofactors is crucial for the biotransformation efficiency [20,21]. Simultaneous overexpression of target enzymes and NAD(P)H regeneration by enzymes in whole-cell biocatalysts has been carried out in many asymmetric reduction systems [22]. Xiao et al. used NAD^+^ regeneration to extend the applications of NAD(P)^+^-dependent oxidoreductases by whole-cell biocatalysis [23]. Cofactor regeneration has been successfully applied *in vitro* for the production of optically active alcohols by the introduction of glucose dehydrogenase (GDH) [24,25]. Kataoka et al. reported a system consisting of carbonyl reductase coupled with an NADPH regenerating system comprising GDH [26–28]. Gröger et al. developed a highly efficient tailor-made “designer cells” for the desired asymmertric reduction by coexpressing the corresponding alcohol dehydrogenase with GDH enzyme [9]. 

In our previous work, we showed that *C. parapsilosis* SCR II catalyzes β-hydroxyacetophenone to (*S*)-PED [6,7]. The enzymatic biosynthesis requires NADPH as a cofactor. However, when the substrate concentration of β-hydroxyacetophenone was higher than 6 g/L, the biotransformation efficiency was not satisfactory. The cause may be insufficient cofactors or an equivalent imbalance between the enzyme and cofactor during the reduction [29]. Furthermore, the enzyme SCRII exhibits a narrow substrate spectrum [30], and has almost no catalytic function for β-hydroxyacetophenone derivatives. In this work, to satisfy the growing demand for novel reductive biocatalysts, we focus on the redesigning new function of SCRII by site mutation in or adjacent to the substrate-binding pocket located between αF and αFG [14,30]. The mutants altered the enantiopreference and inverted the enantioselectivity during the bioreduction of acetophenone to (*R*)-phenylethanol (PE). We introduced *Bacillus* sp. YX-1 GDH into the pathway of (*R*)-PE in *Escherichia coli*, and then optimized the SCRII and GDH coexpression system by altering the orders of their coding genes and using the different linkers between them. The newly designed coexpression system not only altered the substrate specificity, but also improved the enantioselective behavior of the desired enzyme. The system stimulated significant increases in acetophenone reductive activity and the yield of the product, (*R*)-PE, while simultaneously reducing the reaction time from 48 h to 1 h, when 0.06 mM NADP^+^ was added at the beginning of the reaction.

## Materials and Methods

### Organisms and chemicals

*Bacillus* sp. YX-1 was used as a GDH coding gene (*gdh*) donor. The strain was cultured in Luria Broth medium at 37 °C for 16 h. The restriction enzymes were purchased from the Takara Shuzou Co. (Kyoto, Japan). The substrates, i.e., β-hydroxyacetophenone and acetophenone were purchased from the Sigma-Aldrich Chemical Co. Inc. All other chemicals were of the highest grade that could be obtained commercially.

### Site-directed mutagenesis of SCRII

The coding gene of SCRII (*scr*II, GenBank ID: GQ411433) was expressed in *E. coli* as described by Zhang et al. [30]. The Ala220Asp and Glu228Ser mutants were generated using a modified overlap-extension technique [31] with pET-SCRII as the template [30]. The plasmids pET-A220D and pET-E228S containing A220D and E228S mutations were obtained and verified by DNA sequencing. The primer pairs used are listed in Table S1 in File S1.

### Gene cloning of GDH

The *gdh* gene was cloned from *Bacillus* sp. YX-1 using the degenerate primers (*gdh*_F1: TTYGGNACNCTNGAYRTNATGTA and *gdh*_R1: CCNATRTANCCCATNGGNATCAT). Since the amplified DNA of *gdh* shares high similarity with *B. amyloliquefaciens* DSM7, the primers [*gdh*_F2:GAATTCCATATGTAC (*Nde*I) and *gdh*_R2:ATCCTGAGCTCTTATCCGCGGCC (*Sac*I)] were designed to clone the whole gene. The *gdh* gene was inserted on the pET-28(a)^+^ to obtain the recombinant plasmid pET-GDH.

### Construction and optimization of coexpression system

Several coexpression systems containing the E228S and GDH mutants were constructed using two different linkers between them. One was a flexible linker (GGGGS)_3_, the other a Shine–Dalgarno (SD) and aligned spacing (AS) sequence (Figure S1 in File S1). Either E228S or GDH was nearest to the promoter. When the flexible linker (GGGGS)_3_ was used, the other two fusion genes E228S-(GGGGS)_3_-GDH (MS-L-G) and GDH -(GGGGS)_3_- E228S (G-L-MS) were cloned using a modified overlap-extension technique [31]. When the SD-AS sequence was used as the linker, the two fusion genes E228S-SD-AS-GDH (MS-SD-AS-G) and GDH-SD-AS-E228S (G-SD-AS-MS), were generated using an overlap-extension technique [31] In each of the fusion genes, the leftmost genes were nearest the promoter. Then the four fusion genes were constructed on the plasmid pET-28(a)^+^, and the corresponding recombinant plasmids, pET-MS-L-G, pET-G-L-MS, pET-MS-SD-AS-G and pET-G-SD-AS-MS were obtained by DNA sequencing. The primer pairs used are listed in Table S1 in File S1.

### Protein purification

All the recombinant proteins were expressed in *E. coli* strain BL21 (DE3) as His_6_-tagged proteins. First, the protein was purified by affinity chromatography on an Ni^2+^-Sepharose column (His-Trap Kit, Pharmacia). Second, the pooled fractions were further loaded onto a Resource Q column (1 cm × 1 cm) equilibrated with a buffer (20 mM Tris-HCl, pH 8.5) with an ÄKTA Protein Purifier system (Pharmacia, Uppsala, Sweden). Finally, the fraction was applied to a Superdex 200 column for chromatography in a buffer containing 20 mM Tris-HCl (pH 8.5) and 150 mM NaCl. 

### Enzyme assay

The enzymatic activities of SCRII and its variants for the reduction of β-hydroxyacetophenone or acetophenone were measured at 35 °C by recording the rate of change in the NAD(P)H absorbance at 340 nm, as described by Zhang et al. [30]. The assay mixture for the GDH activity contained 100 mM Tris-HCl (pH 8.0), 100 mM glucose, and 2 mM NADP^+^, and the reactions at 30 °C were monitored as the increase in absorbance at 340 nm. One unit of reductase (SCRII or its variants) activity or GDH activity is defined as the amount of enzyme catalyzing the oxidation of 1 μmol NADPH or the reduction of 1 μmol NAD(P)^+^ under the measurement condition, respectively. Protein concentrations were determined using a Bio-rad protein assay kit using bovine serum albumin as a standard [32].

### Determination of kinetic parameters

The kinetic parameters of SCRII and its variants were determined using various concentrations of β-hydroxyacetophenone (0.5–20 mM) or acetophenone (0.5–20 mM) substrate, enzyme (10–200 μM), and cofactors NADPH (0.5–5.0 mM) in 100 mM phosphate buffer (pH 7.5) [30]. The data were fitted to the Michaelis–Menten equation by a nonlinear least-squares iterative method using KaleidaGraph (Synergy Software, Reading, PA, USA). Three sets of kinetic parameters were obtained from three independent experiments and then averaged to yield the final estimates. The final estimates are shown with the standard errors for the three sets.

### Biotransformation and analytical methods

The biotransformation reactions were carried out as described previously [6] with minor modifications. For the asymmetric reaction with the recombinant *E. coli* cells, the reaction mixture (2 mL) consisted of 0.1 M potassium phosphate buffer (pH 6.5), 10 -20 g/L β-hydroxyacetophenone or acetophenone, 40 g/L glucose, and 0.2 g washed wet cells. When the purified protein was used as the biocatalyst, the reaction mixture (2 mL) consisted of 0.1 M potassium phosphate buffer (pH 6.5), 0.005–0.5 mM NADH or NADPH, 10 g/L β-hydroxyacetophenone or acetophenone, 40 g/L glucose, and an appropriate amount of purified protein. The reactions were carried out at 30 °C for 20 h with shaking at 200 rpm, using the wet recombinant cells and purified enzyme as biocatalysts. At the end of the reaction, i.e., the product (*S*)-PED or (*R*)-PE, was extracted with ethyl acetate, and the organic layer was used for analysis. The optical purity and yield of the product were determined using high-performance liquid chromatography on a Chiralcel OB-H column (Daicel Chemical Ind. Ltd., Japan). The retention times of (*S*)-PED and (*R*)-PE are 23.5 and 13.8 minutes, respectively.

### Determination of intracellular nucleotide concentrations

The extraction of intracellular nucleotides was carried out as previously described by Nissen et al. [33], with minor modifications. The collected recombinant *E. coli* (5.0 mL) culture was mixed with 20 mL of 60% methanol (−40 °C) within 1 s. A 50 mM potassium phosphate buffer (pH 5.0) and 50 mM Tris–HCl (pH 9.0) were used for extraction of NADP^+^ and NADPH, respectively. The nucleotide concentrations were measured immediately after reducing the sample volumes by evaporation under vacuum (30 min, 5 °C). The NADP^+^ and NADPH contents of the samples were determined as described by Bergmeyer [34], using standard curves for each compound. Assays were performed in triplicate.

### Protein structure homology modeling

On the basis of a sequence alignment between the protein SCRII and the template structure SCR (PDB ID: 3CTM), a three-dimensional model for SCRII is generated using SWISS-MODEL workspace (http://swissmodel.expasy.org//SWISS-MODEL.html).

### Autodocking

Docking procedures were carried out according to the methodology described in the program ICM v.3.4-9d [35]. The protein structure of SCRII was used to provide receptors. The new conformation was accepted or rejected according to the metropolis criterion, using a temperature of 600 K. Each random change was followed by 100 local conjugate-gradient minimization steps against grid maps. The length of the docking run and the length of local minimization was determined automatically by an adaptive algorithm, depending on the size and number of flexible torsions in the ligand. Visual inspection was performed for the lowest energy conformations satisfying the absence of clashes after docking.

## Results and Discussion

### Enantioselectivity alteration of SCRII by site-mutagenesis

**Figure 1:**
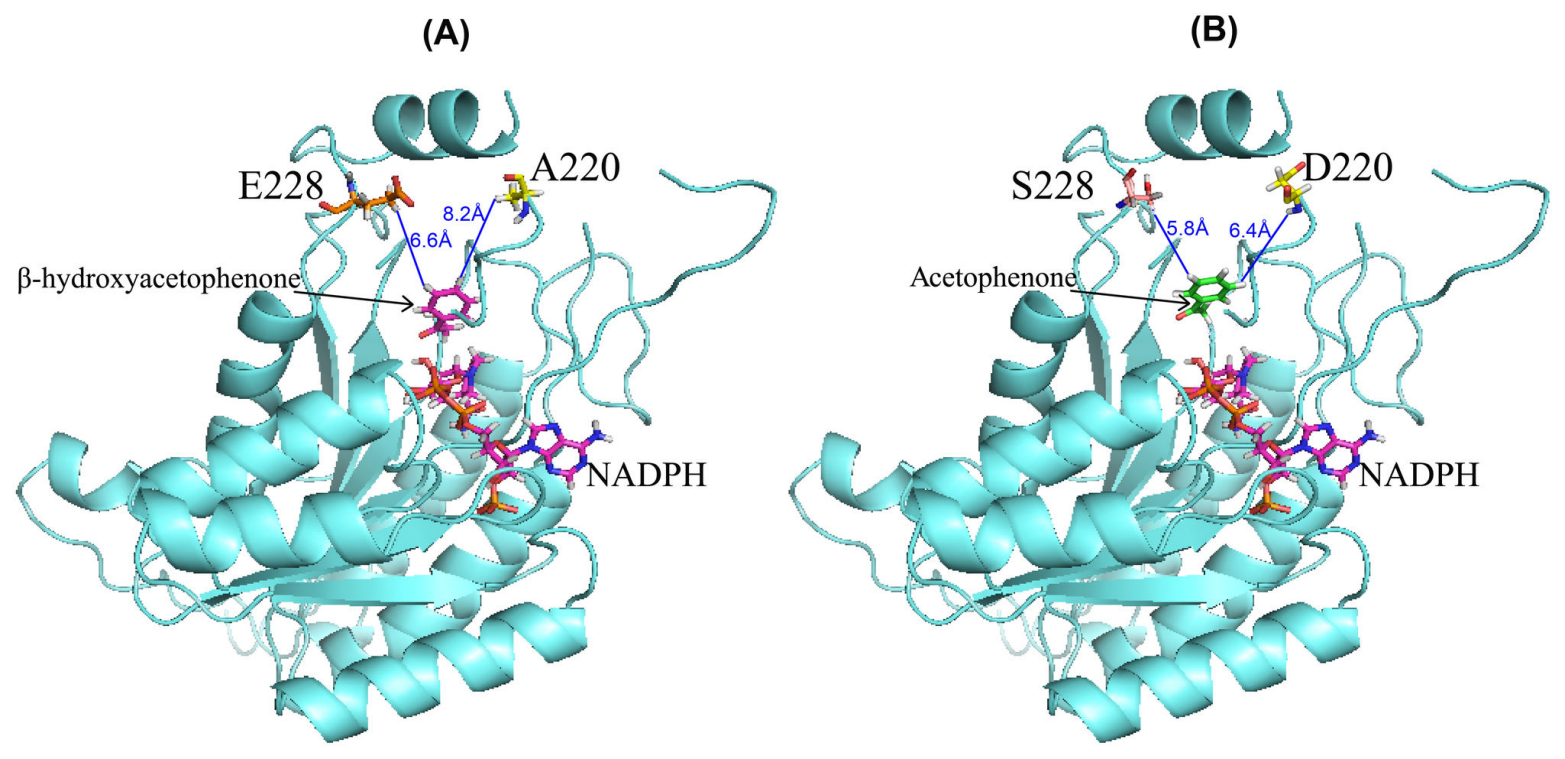
Effect of A220D and E228S on enzyme activity. (A) Wild type enzyme; (B) A220D and E228S mutants. A220 and E228 are both located on the entrance or channel of substrate-binding loop. The molecular graphics image was drawn using PyMOL software.

It has been reported that wild-type (WT) SCRII from *C. parapsilosis* catalyzes an anti-Prelog reduction of β-hydroxyacetophenone to (*S*)-PED [30]. Based on the sequence-structure alignment, we designed several single-point mutations inside or adjacent to the substrate-binding loop or active site, which located at distal to the αF helix [7,14,30]. The data in Table 1 show that the mutations A220D and E228S almost lost enantioselectivity towards β-hydroxyacetophenone reduction. The specific activities of the A220D and E228S variants for β-hydroxyacetophenone reduction decreased about 10- fold and 11-fold, respectively, and their yields towards (*S*)-PED were both reduced by 9.6-fold and 11.8-fold with respect to the WT SCRII (Table 1). These results suggested A220 and E228 are important in substrate-binding and/or catalysis [30]. The variants A220D and E228S were further tested for acetophenone reductive activity by following the rate of NADPH consumption in time at 340 nm. They showed increases in enantiomeric activities for acetophenone of about 10-fold and 22-fold, respectively, compared with WT SCRII (Table 1). When the whole recombinant cells were used as catalysts, the variants A220D and E228S produced (*R*)-PE of optical purity of ≥99% in acetophenone reduction. The yields of (*R*)-PE were 43.4% and 80.2%, which were over 14-fold and 25 times, respectively, those obtained with the WT enzyme (Table 1). The A220D and E228S mutations resulted in significant attenuation of (*S*)-PED selectivity in β-hydroxyacetophenone reduction, but they showed significant increases in (*R*)-PE enantioselectivity during acetophenone reduction. So the A220D and E228S mutants altered the enantioselectivity towards (*R*)-PE in the acetophenone reduction. The SCRII structure model was obtained by homology modeling based on the X-ray structure of SCR (PDB code: 3CTM) [7]. Based on the highly conserved cofactor-binding domain, putative enzyme-cofactor-substrate docking was performed with β-hydroxyacetophenone or acetophenone and NADPH, respectively (Figure 1). Lowest-energy conformational ensembles show that structure comparison suggested that the steric conformation in the substrate binding domain is more favorable to acetophenone with A220D and E228S. The residues Ala220 and Glu228 are both located in the entrance or channel of substrate binding loop. In Figure 1, the distance between A220 or E228 of WT enzyme and benzene ring of β-hydroxyacetophenone (from 6.6 to 8.2 Å) is greater than that of D220 or S228 of mutants and benzene ring of acetophenone (from 5.8 to 6.8 Å). This situation may cause the WT SCRII to show the substrate enantioselectivity of acetophenone >>β-hydroxyacetophenone [36]. When A220 is substituted by Asp or E228 is substituted by Ser, the hydrophobic interaction between the substrate binding pocket and acetophenone is stronger, causing increased efficiency of acetophenone 1″-hydroxylation comparable to that of β-hydroxyacetophenone 1″-hydroxylation. These results suggested that the enzyme subtle structure of has a significant influence on the enzyme activity and affinity towards substrates [37].

**Table 1:** Asymmetric reductions of β-hydroxyacetophenone and acetophenone catalyzed by SCRII and its variants.

	**SCRII–WT ^a^**			**SCRII–A220D ^a^**			**SCRII–E228S ^a^**		
**Substrates**	**Specific activity (U/mg)**	**Yield (%)**	**Optical purity (%)**	**Specific activity (U/mg)**	**Yield (%)**	**Optical purity (%)**	**Specific activity (U/mg)**	**Yield (%)**	**Optical purity (%)**
β-Hydroxyacetophenone	5.8±0.2	86.1±3.4	≥ 99(*S*)	0.6±0.1	9.0±0.7	≥ 99(*S*)	0.5±0.1	7.3±0.2	≥ 99(*S*)
Acetophenone	1.1±0.4	3.2±0.3	≥ 99(*R*)	11.2±0.5	43.4±2.2	≥ 99(*R*)	24.3±0.1	80.2±3.8	≥ 99(*R*)

Notes: ^a^ Recombinant *E. coli* BL21 (DE3) was cultured until the turbidity of culture at 600 nm reached 0.6 to 0.8, and cells were cultured for another 8 h after the addition of 1 mM IPTG at 20°C.

### Increased catalytic efficiency by E228S mutant in acetophenone reduction

The kinetic effects of the mutations on the directions of β-hydroxyacetophenone and acetophenone reduction with WT SCRII and its variants were assessed. The kinetic parameters were determined for β-hydroxyacetophenone or acetophenone at different concentrations (0.5–20 mM) and cofactors at various concentrations (0.5–5 mM). The kinetic results are shown in Table 2. In the reduction of β-hydroxyacetophenone, the *K*_M_ values of the A220D and E228S variants were both increased about 2-fold, whereas their *k*_cat_ values were decreased 7.8-fold and 12.9-fold, respectively, with respect to WT SCRII. The A220D and E228S mutations resulted in 26.3-fold and 15.5-fold reductions in the *k*_cat_/*K*_M_ values with β-hydroxyacetophenone. Their increased *K*_M_ and decreased *k*_cat_/*K*_M_ values (Table 2) resulted in a low biotransformation efficiency towards (*S*)-PED in β-hydroxyacetophenone reduction. For acetophenone reduction, the A220D and E228S variants showed lower *K*_M_ values but higher *k*_cat_ values compared with WT SCRII. The A220D and E228S mutations resulted in nearly 3-fold and 5-fold reductions in the *K*_M_ values, but 2.9-fold and 4.6-fold increase in the *k*_cat_ values compared with the WT enzyme. The most striking change resulting from the mutagenesis is that the *k*_cat_/*K*_M_ ratio between A220D and WT was 7.3, and the ratio between E228S and WT was 20.8 for acetophenone reduction. These results show that the catalytic efficiency with acetophenone of the E228S variant is much higher than those of WT SCRII and its A220D variant. The E228S mutant showed the highest biotransformation efficiency during acetophenone bioreduction among the WT enzymes and its variants. Structural analysis showed that the distances between the residues A220 or E228 and catalytic groups are less than 20 Å in the *apo*-strucutre of SCR II [30]. The single mutations can propagate structural changes to the active site, where they cause subtle structural changes, resulting in improved enantioselectivity in acetophenone reduction [15,38]. These results indicate that the A220D and E228S variants not only favor acetophenone over β-hydroxyacetophenone as the substrate, but also alter the enantioselective behaviors of the WT enzymes and the product enantioselectivity during the reaction: from (*S*)-PED (in β-hydroxyacetophenone reduction) to (*R*)-PE (during acetophenone reduction). Moreover, the E228S mutant had higher catalytic efficiency for acetophenone reduction than the A220D mutation did. Therefore, the E228S mutant was therefore selected for further construction of a coexpression system to improve (*R*)-PE production.

**Table 2:** Kinetic parameters for β-hydroxyacetophenone and acetophenone reductions by the WT SCRII and its variants.

	**SCRII–WT**			**SCRII–A220D**			**SCRII–E228S**		
**Substrates**	***K*_M_ (μM)**	***k*_cat_ (S^-1^)**	***k*_cat_/*K*_M_**	***K*_M_ (μM)**	***k*_cat_ (S^-1^)**	***k*_cat_/*K*_M_**	***K*_M_ (μM)**	***k*_cat_ (S^-1^)**	***k*_cat_/*K*_M_**
			**(×10^6^ S^-1^ M^-1^)**			**(×10^6^ S^-1^ M^-1^)**			**(×10^6^ S^-1^ M^-1^)**
β-hydroxyacetophenone	4.58 ± 0.17	15.66 ± 0.19	3.42	9.52 ± 0.09	1.21 ± 0.08	0.13	9.20 ± 0.11	2.02 ± 0.14	0.22
acetophenone	15.47±0.31	2.73±0.02	0.18	5.91±0.29	7.82±0.06	1.32	3.38±0.11	12.64±0.26	3.74

Notes: All reactions involved in the calculation of kinetic constants calculation were assayed at 100 mM acetate buffer (pH 5.0) and 35°C. All experiments were repeated three to five times.

### Coexpression of E228S and GDH

In any system based on carbonyl reductase catalyzing the bioconversion of a chiral alcohol, the insufficient cofactors or the stoichiometrically unbalanced ratios between the enzyme and the cofactor result in low biotransformation efficiency [39–41]. During metabolism, a cell oxidizes a carbon source, such as glucose, using NADP^+^ as the cofactor, producing reducing equivalents in the form of NADPH. The cell regenerates NADPH from NADP^+^ to achieve a redox balance. Heterogeneous expression of GDH is expected to increase the availability of NADPH in the whole metabolic network, thus improving the flux of NADPH–dependent pathways [42]. In this work, GDH from *Bacillus* sp. YX-1 was introduced into the metabolic flux of a chiral alcohol with the mutant E228S.

**Figure 2:**
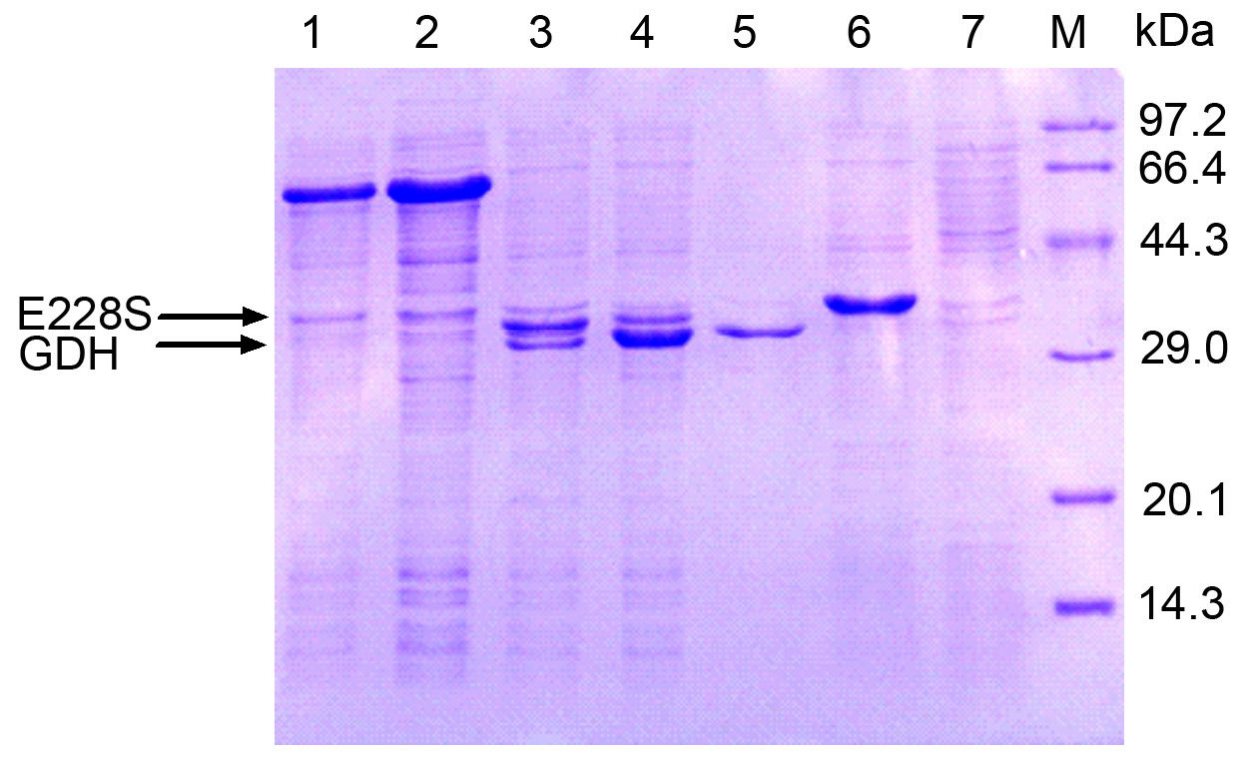
SDS-PAGE analysis of cell extracts of E. **coli transformants**. Lane 1, *E*. *coli*/pET-MS-L-G; Lane 2, *E*. *coli*/pET-G-L-MS; Lane 3, *E*. *coli* /pET-MS-SD-AS-G; Lane 4, *E*. *coli*/pET-G-SD-AS-MS; Lane 5, *E*. *coli*/pET-GDH; Lane 6, *E*. *coli*/pET-E228S; Lane 7, *E*. *coli*/pET-28a; M, molecular mass markers. The gel was stained for protein with Coomassie Brilliant Blue R-250．

Cloning of the GDH gene and construction of coexpression plasmids were performed using the standard techniques described in the Materials and Methods section. The coexpression plasmids obtained were designated as pET-MS-L-G, pET-G-L-MS, pET-MS-SD-AS-G and pET-G-SD-AS-MS, as shown in Figure S1 in File S1. They were then transformed into competent *E. coli* BL21 (DE3) cells, using standard techniques. The recombinant strains *E. coli*/pET-MS-L-G, *E. coli*/pET-G-L-MS, *E. coli*/pET-MS-SD-AS-G and *E. coli*/pET-G-SD-AS-MS were shaped after verification by DNA sequencing. The *E. coli* BL21 cells carrying expression plasmids were induced with 0.1 mM isopropyl-β-thiogalactopyranoside (IPTG) at 17 °C. By Sodium dodecyl sulfate-polyacrylamide gelelectroporesis (SDS-PAGE) analysis showed that in cell-free extracts of *E. coli*/pET-MS-L-G and *E. coli*/pET-G-L-MS, only one predominant band, corresponding to the theoretical size (65 kDa) of the fusion protein of E228S and GDH was observed (Figure 2). These results suggest that the protein E228S and GDH are fused when they are linked by a flexible linker (GGGGS)_3_ in *E. coli*. In cell-free extracts of *E. coli*/pET-MS-SD-AS-G and *E. coli*/pET-G-SD-AS-MS (Figure 2), two obvious bands (at about 30 kDa and 35 kDa) corresponding to the sizes of E228S [30] and GDH [43] were observed. However, the amount of enzyme E228S produced in *E. coli*/pET-MS-SD-AS-G was larger than that in *E. coli*/pET-G-SD-AS-MS; the protein GDH was more highly expressed in *E. coli*/pET-G-SD-AS-MS than in *E. coli* /pET-MS-SD-AS-G. These results show that the expression levels of the target proteins (E228S and GDH) were significantly influenced by altering the order of their coding genes on the plasmid [44]. If either E228S or GDH is moved closer to the promoter, it exhibits a higher protein expression level [43]. Figure 2 also shows that the expression level of the first enzyme gene on the plasmid (upstream) showed little difference whether it was coexpressed or expressed alone, consistent with several previous reports [28,45]. Since the SD and AS sequences initiated translation efficiently [46,47], the enzymes E228S and GDH were highly coexpressed in *E. coli*. The expressed proteins in soluble form allowed further examination of the effects of different coexpression systems on biotransformation efficiency. 

### Rebalancing of intracellular NADP^+^ and NADPH by introduction of GDH

GDH was introduced into the coexpressed *E. coli* in the upstream or downstream of the plasmid, and we measured the intracellular concentrations of NADP^+^ and NADPH, and their ratios, in the recombinant cells in the exponential growth phase. The data obtained are summarized in Table 3. There is no obvious differences in total NADP(H) concentrations among the three strains, CK, *E. coli*/pET-MS-SD-AS-G and *E. coli*/pET-G-SD-AS-MS, or their early and late exponential growth phases (data not shown), the NADP^+^ and NADPH concentrations differed in the different *E. coli* strains. The presence of GDH increased the NADPH concentration and decreased the NADP^+^ pool compared with CK, resulting in an increase in the NADPH/NADP^+^ ratio. In the recombinant strain *E. coli*/pET-MS-SD-AS-G, in which GDH gene was located in the downstream of the plasmid, the NADPH/NADP^+^ ratio was 12.29, with an increase of 2.3-fold, compared with that for CK. GDH downstream of pET-G-SD-AS-MS stimulated an 4.1-fold increase in the the NADPH/NADP^+^ value. These results show that the introduction of GDH did not change the total concentrations of NADP(H), but their ratios were redistributed in the engineered *E. coli*. NADP^+^ is efficiently converted to NADPH by the GDH. GDH upstream is more conducive to the release of NADPH than is GDH downstream. The increased NADPH concentration and NADPH/NADP^+^ ratio would be advantageous in reduction catalyzed by NADP^+^-dependent SCRII. The cofactor rebalancing was expected to contribute to the biosynthesis of the final product.

**Table 3:** Intracellular concentrations of NADP^+^ and NADPH in recombinant cells during exponential growth.

	**Intracellular concentrations (μmol/g [dry wt] of biomass) of:**			
**Strains**	**NADP^+^**	**NADPH**	**Total**	**NADPH/NADP^*+*^ ratio**
CK	0.14±0.01	0.76±0.06	0.90±0.08	5.42±0.04
*E. coli* /pET-MS-SD-AS-G	0.07±0.01	0.86±0.05	0.93±0.07	12.29±0.06
*E. coli* /pET-G-SD-AS-MS	0.04±0.02	0.91±0.07	0.95±0.03	22.75±0.09

### Rebalancing functions of E228S and GDH by alteration of gene orders on plasmid

The specific enzyme activities were determined in the coexpression systems at the exponential growth phase. The results are summarized in Table 4. When a flexible linker (GGGGS)_3_, was inserted between E228S and GDH, the reductase activity and GDH activity were 2.7 U/mg and 6.7 U/mg, and the ratio of reductase activity/GDH activity was 0.4 in the strain *E. coli*/pET-MS-L-G. However, no enzyme activities were detected in any fractions of *E. coli*/pET-G-L-MS. Even when the flexible linker (GGGGS)_3_ was replaced by a longer or shorter flexible linker, or was changed to a rigid one, specific activities were not found in the strain *E. coli*/pET-MS-L-G (data not shown). It is important to note that the linker apparently does not allow the catalytic domains of E228S and GDH to align properly and undergo oligomerization in *E. coli*/pET-G-L-MS [48,49]. When the SD-AS sequence was used as the linker between E228S and GDH, the *E. coli*/pET-MS-SD-AS-G exhibited an activity of 8.2 U/mg with acetophenone as a substrate, and 7.6 U/mg with glucose as a substrate. The ratio of reductase activity to GDH activity was 1.08. In the cell-free of *E. coli*/pET-G-SD-AS-MS, the reductase activity and GDH activity were 1.1 U/mg and 53.6 U/mg respectively, and their ratio was reduced to 0.02. When the E228S gene was moved from upstream (in *E. coli*/pET-MS-SD-AS-G ) to downstream (in *E. coli*/pET-G-SD-AS-MS ) of the promoter on the plasmid, the reductase activity decreased 7.5-fold, whereas the GDH activity increased 7.1-fold. These results suggest that the distance between the genes and promoters have a significant effect on the protein expression levels and enzyme activities. The two target enzymes, E228S and GDH, were both functionally expressed in *E. coli*, and their activities were rebalanced by altering their gene orders on the plasmid, probably resulting in metabolic flux redistribution in the (*R*)-PE pathway [44]. 

**Table 4:** Reductase and GDH activities in cell-free extracts of recombinant *E. coli.*

	**Enzyme activities ^a^**		**Biotransformation ^c^**	
**Recombinant *E. coli***	**Reductase activities (U/mg)**	**GDH activities (U/mg)**	**Yield (%)**	**e.e. (%)**
*E. coli* /pET-MS-L-G	2.7±0.1	6.7±0.5	17.4±0.7	99.4±1.7
*E. coli* /pET-G-L-MS	– ^b^	–	–	–
*E. coli* /pET-MS-SD-AS-G	8.2±0.8	7.6±0.3	92.2±0.2	99.5±1.2
*E. coli* /pET-G-SD-AS-MS	1.1±0.1	53.6±0.2	3.5±0.7	99.5±0.6

Notes: ^a^ The reductase and GDH activities mean the special activities per milligram of wet cells; ^b^ – means no enzyme activity or no chiral alcohol was detected.

^c^ The biotransformation was carried out at pH 6.5, 35°C with 10 g/L acetophenone as substrate.

### Efficient biotransformation of (R)-PE by E. coli /pET-MS-SD-AD-G

**Figure 3:**
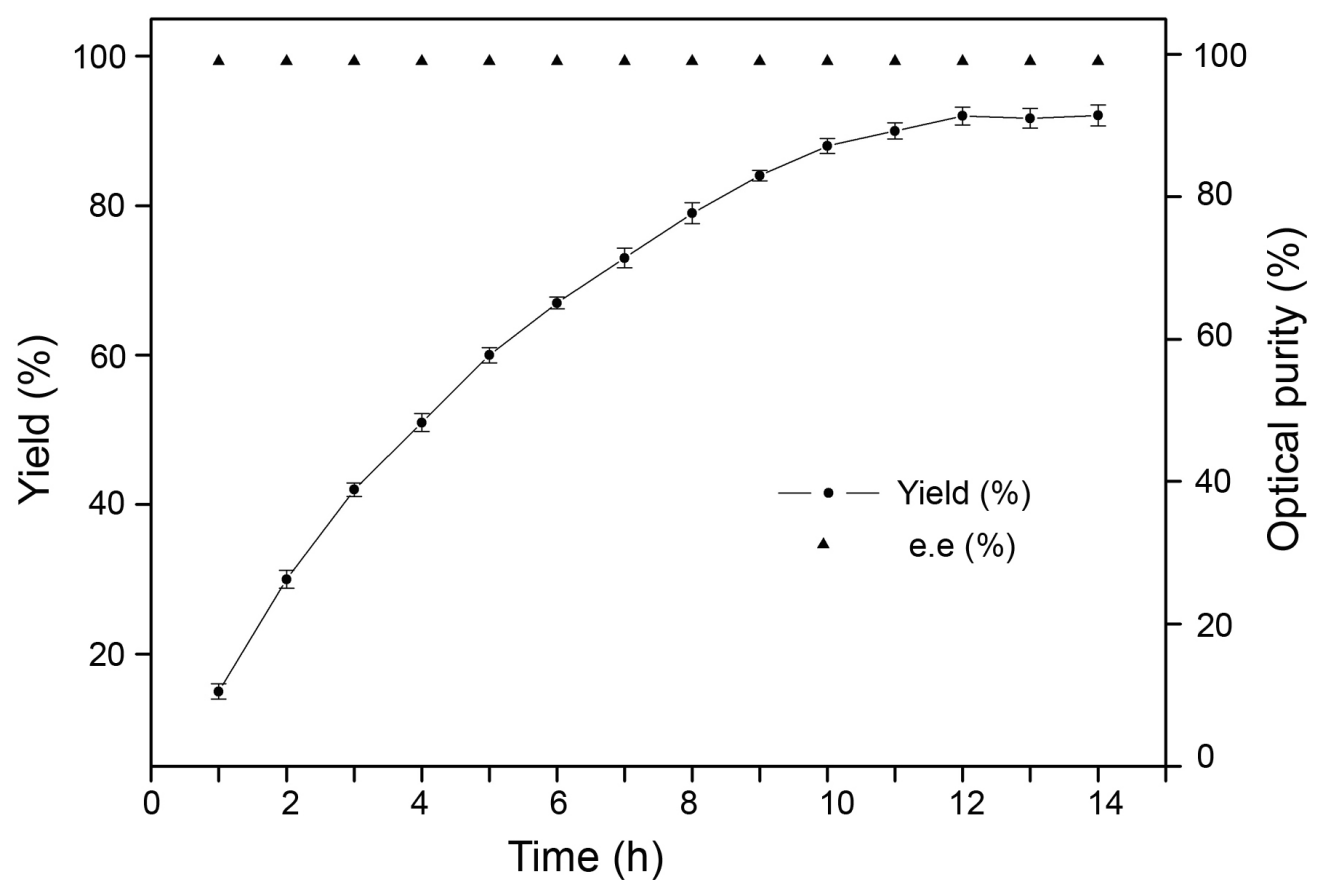
Time courses of asymmetric reduction of 10 g/L acetophenone by *E*. ***coli*/pET-MS-SD-AS-G**. Error bars represent standard deviations (*n* = 3).

The effect of altering the gene order on the rebalancing the enzyme functions in (*R*)-PE biotrasformation was examined when whole cells of the coexpression systems were used as catalysts. Since the GDH activity is higher than the reductase activity of E228S in *E. coli*, the biotransformation was investigated at the optimal for SCRII, i.e., pH 6.5 [30,50]. The optical purity of PE obtained catalytically from acetophenone was detected by high-performance liquid chromatography, using a chiral column. Samples were taken and analyzed with respect to the (*R*)-PE formation at certain times. The results (Figure S2 in File S1 and Table 5) showed that (*R*)-PE was formed by all the coexpression systems, with different efficiencies, except *E. coli*/pET-G-L-MS. When using 10 g/L of acetophenone as the substrate, no chiral alcohols were detected in the reaction mixture with *E. coli* /pET-G-L-MS. The other systems produced (*R*)-PE of optical purity over 99%, but in different yields: the strains *E. coli*/pET-MS-L-G and *E. coli*/pET-G-SD-AS-MS produced (*R*)-PE in very low yields (3.5% and 17.4%, respectively), and the *E. coli*/pET-MS-SD-AS-G strain showed a good performance, giving (*R*)-PE in a high yield of 92.2% (Figure 3). Furthermore, the asymmetric reaction with *E. coli*/pET-MS-SD-AS-G proceeded very quickly and reached the highest point at 12 h (Figure 3). Compare with the *E. coli*-SCRII, the reaction time decreased 4-fold [30], suggesting that GDH introduction into the SCRII pathway accelerated the bioconversion of acetophenone to (*R*)-PE. Compared with previous reactions using *E. coli*-SCRII alone, the single site-mutation and the introduction of GDH yielded increased the substrate concentration 2-fold [30]. Based on these results, it was suggested that the coexpression system *E. coli* /pET-MS-SD-AS-G catalyzed stereospecific reduction of acetophenone, and produced (*R*)-PE efficiently. 

**Table 5:** Asymmetric reduction of acetophenone by recombinant *E. coli* strains.

**Substrate concentrations (g/L)**	***E. coli* /pET-MS-L-G**		***E. coli* /pET-G-L-MS**		***E. coli* /pET-MS-SD-AS-G**		***E. coli* /pET-G-SD-AS-MS**	
	**e.e. (%)**	**Yield (%)**	**e.e. (%)**	**Yield (%)**	**e.e. (%)**	**Yield (%)**	**e.e. (%)**	**Yield (%)**
10	99.4±1.7	17.4±0.7	–	–	99.5±1.2	92.2±0.2	99.5±0.6	3.5±0.7
15	99.8±2.3	7.3±0.3	–	–	99.3±2.0	57.7±1.1	99.7±0.9	1.3±0.1
20	99.4±3.1	2.5±0.2	–	–	99.7±1.7	30.9±0.9	99.4±1.8	0.7±0.1

Notes: “–” means no chiral alcohol was detected.

As a complementary approach, the biotransformation was performed using cell-free exacts from *E. coli*/pET-MS-SD-AS-G to reduce acetophenone in the presence of NADP^+^. The results showed that the crude enzymes of *E. coli*/pET-MS-SD-AS-G produced (*R*)-PE in a higher yield than its corresponding transformants did. Different concentrations of the cofactor affected on the stereoselectivity of product. When 0.06 - 0.5 mM NADP^+^ was added to the reaction mixture, the cell-free of *E. coli*/pET-MS-SD-AS-G showed an excellent performance, giving (*R*)-PE of high optical purity, above 99%, and a high yield of 92-93% in 1 h (Table 6). The biosynthesis performance in the production of (*R*)-PE in the presence of 0.06 mM NADP^+^ was essentially the same, but the reaction time increased to 2 h (Table 6). When the NADP^+^ concentration was lower than 0.03 mM, the (*R*)-PE yield was not satisfactory. So, when NADP^+^ of concentration greater than 0.06 mM was added as the substrate for GDH at the beginning of the reaction, cofactor regeneration was more efficient, resulting in significantly shorter times, from 48 h to 1 h, with coexpressed *E. coli*. Adding a certain amount of NADP^+^ to the E. coli/pET-MS-SD-AS-G lysate shortened the reduction time, and increased the yield significantly, which may be due to the stronger binding ability between the crude enzyme and the substrate than between the cells and the substrate. Moreover, the first added NADP^+^ was used as the substrate for GDH, which can accelerate *in situ* cofactor regeneration by the coexpression system.

**Table 6:** Effects of coenzyme concentrations on acetophenone reduction by cell-free extracts of *E*. *coli*/pET-MS-SD-AS-G.

**Concentration of NADP^+^ (mM)**	**e.e. (%)**	**Yield (%)**	**Reaction time (h)**
0.5	99.3±0.2	92.8±0.4	1
0.1	99.5±0.1	92.2±0.7	1
0.06	99.3±0.1	92.3±0.5	1
0.03	99.4±0.3	92.0±1.1	2
0.01	99.3±0.2	70.3±1.9	3.5
0.005	99.5±0.1	64.4±2.4	7
0	99.5±0.3	53.2±3.1	12

Notes: The reaction was carried out at the conditions of 10 g/L acetophenone, 35°C, pH 6.5.

## Conclusions

This study demonstrates that it is possible to tailor WT SCRII, using a single mutation to yield a “synzyme”, which alters the enantiopreference of the enzyme and catalyzes acetophenone reduction to produce (*R*)-PE. Then the enzyme GDH was introduced into the pathway of (*R*)-PE with the mutant E228S SCRII for cofactor regeneration. Since the activity of GDH from *Bacillus* sp. YX-1 was higher than that of E228S from *C. parapsilosis*, we minimized the activity levels of the two enzymes when they were coexpressed in *E. coli*. To balance the functions of the two enzymes, the E228S gene was moved closer to the promoter to give a higher expression level and a higher enzyme activity in the coexpression system, thus provoking a significant metabolic redistribution in the (*R*)-PE flux. The optimized coexpression system produced (*R*)-PE with good enantioselectivity: a high optical purity of over 99%, and a high yield of about 92% in 1 h, when 0.06 mM NADP^+^ was added at the beginning of the reaction. With respect to that obtained with WT SCRII, the yield of (*R*)-PE increased 30-fold, and the reaction time was reduced 48-fold. The convenient operation and excellent biosynthesis performance make these microbial biotransformations attractive and practical for the synthesis of highly enantiopure (*R*)-PE. It is hoped that this work will pave a way to engineering SDR catalysts to alter their enantiopreference, and enable the optimization of coexpression system for efficient chiral compound biosynthesis.

## Supporting Information

File S1 **Supporting Information**.
Figure S1. The maps of co-expression plasmids. When the flexible linker was used between the mutant E228S and GDH, the SD-AS sequence was changed by (GGGGS)_3_. Figure S2. Asymmetric reduction of acetophenone using different coexpression systems. (A) Retention times of standard samples are as follows: (*R*)-PE, 13.8 min; (*S*)-PE, 10.8 min; acetophenone, 17.2 min. (B) *E*. *coli*/pET-G-L-MS catalyzed asymmetric reduction of acetophenone; (C) *E*. *coli*/pET-MS-L-G catalyzed asymmetric reduction of acetophenone; (D) *E*. *coli*/pET-G-SD-AS-MS catalyzed asymmetric reduction of acetophenone; (E) *E*. *coli*/pET-MS-SD-AS-G catalyzed asymmetric reduction of acetophenone; AU, arbitrary units. Table S1. Plasmids, strains and primers used
(DOC)
