# Supplementary material for: Efficicent (R)-Phenylethanol Production with Enantioselectivity-Alerted (S)-Carbonyl Reductase II and NADPH Regeneration
Source: PLoS One. 2013 Dec 17;8(12):e83586. doi: 10.1371/journal.pone.0083586 (PMC3866161; doi:10.1371/journal.pone.0083586)
Supplement: File S1 — Supporting Information. Figure S1. The maps of co-expression plasmids. When the flexible linker was used between the mutant E228S and GDH, the SD-AS sequence was changed by (GGGGS)3. Figure S2. Asymmetric reduction of acetophenone using different coexpression systems. (A) Retention times of standard samples are as follows: (R)-PE, 13.8 min; (S)-PE, 10.8 min; acetophenone, 17.2 min. (B) E. coli/pET-G-L-MS catalyzed asymmetric reduction of acetophenone; (C) E. coli/pET-MS-L-G catalyzed asymmetric reduction of acetophenone; (D) E. coli/pET-G-SD-AS-MS catalyzed asymmetric reduction of acetophenone; (E) E. coli/pET-MS-SD-AS-G catalyzed asymmetric reduction of acetophenone; AU, arbitrary units. Table S1. Plasmids, strains and primers used (DOC) [file pone.0083586.s001.doc]

## Efficicent (*R*)-phenylethanol Production with Enantioselectivity-alerted (*S*)-carbonyl ReductaseⅡ and NADPH Regeneration

Rongzhen Zhang^1,2^, Botao Zhang^1,3^ Yan Xu^1,2^*, Yaohui Li^1,2^, Ming Li^1,2^, Hongbo Liang^1,2^, Rong Xiao^4^

**Fig. S1.** The maps of co-expression plasmids. When the flexible linker was used between the mutant E228S and GDH, the SD-AS sequence was changed by (GGGGS)_3_.

**
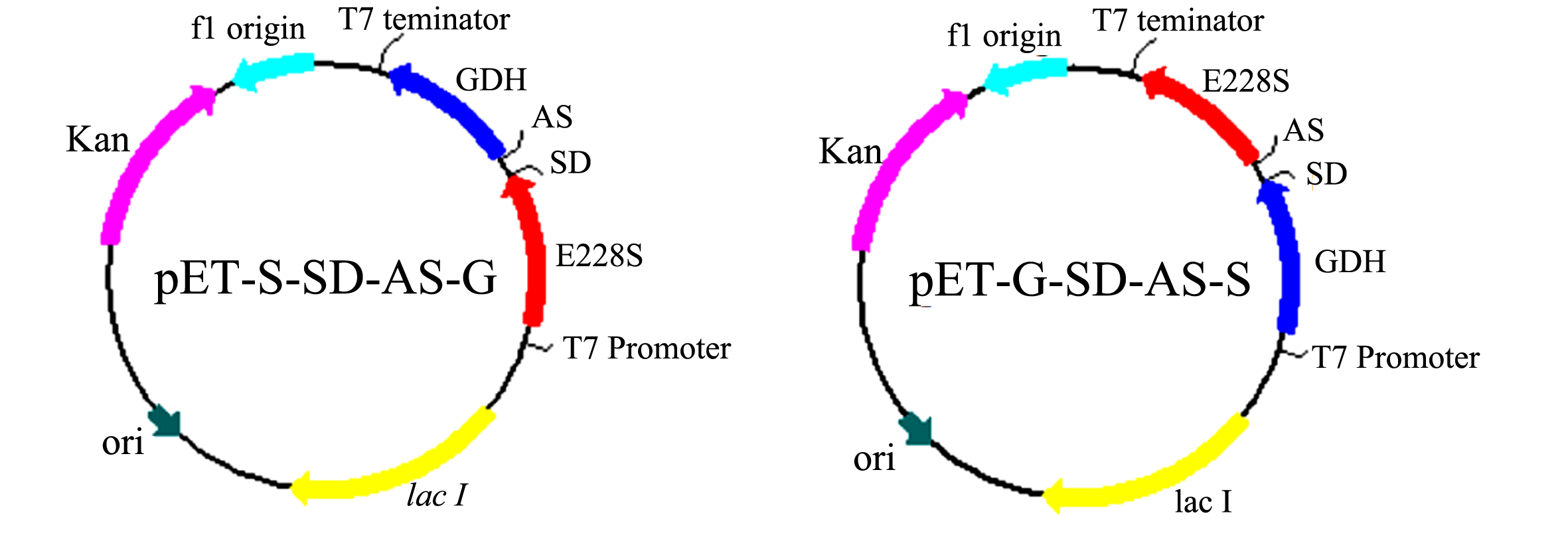
**

**Figure S2.** Asymmetric reduction of acetophenone using different coexpression systems. (A) Retention times of standard samples are as follows: (*R*)-PE, 13.8 min; (*S)*-PE, 10.8 min; acetophenone, 17.2 min. (B) *E. coli*/pET-G-L-MS catalyzed asymmetric reduction of acetophenone; (C) *E. coli*/pET-MS-L-G catalyzed asymmetric reduction of acetophenone; (D) *E. coli*/pET-G-SD-AS-MS catalyzed asymmetric reduction of acetophenone; (E) *E. coli*/pET-MS-SD-AS-G catalyzed asymmetric reduction of acetophenone; AU, arbitrary units.


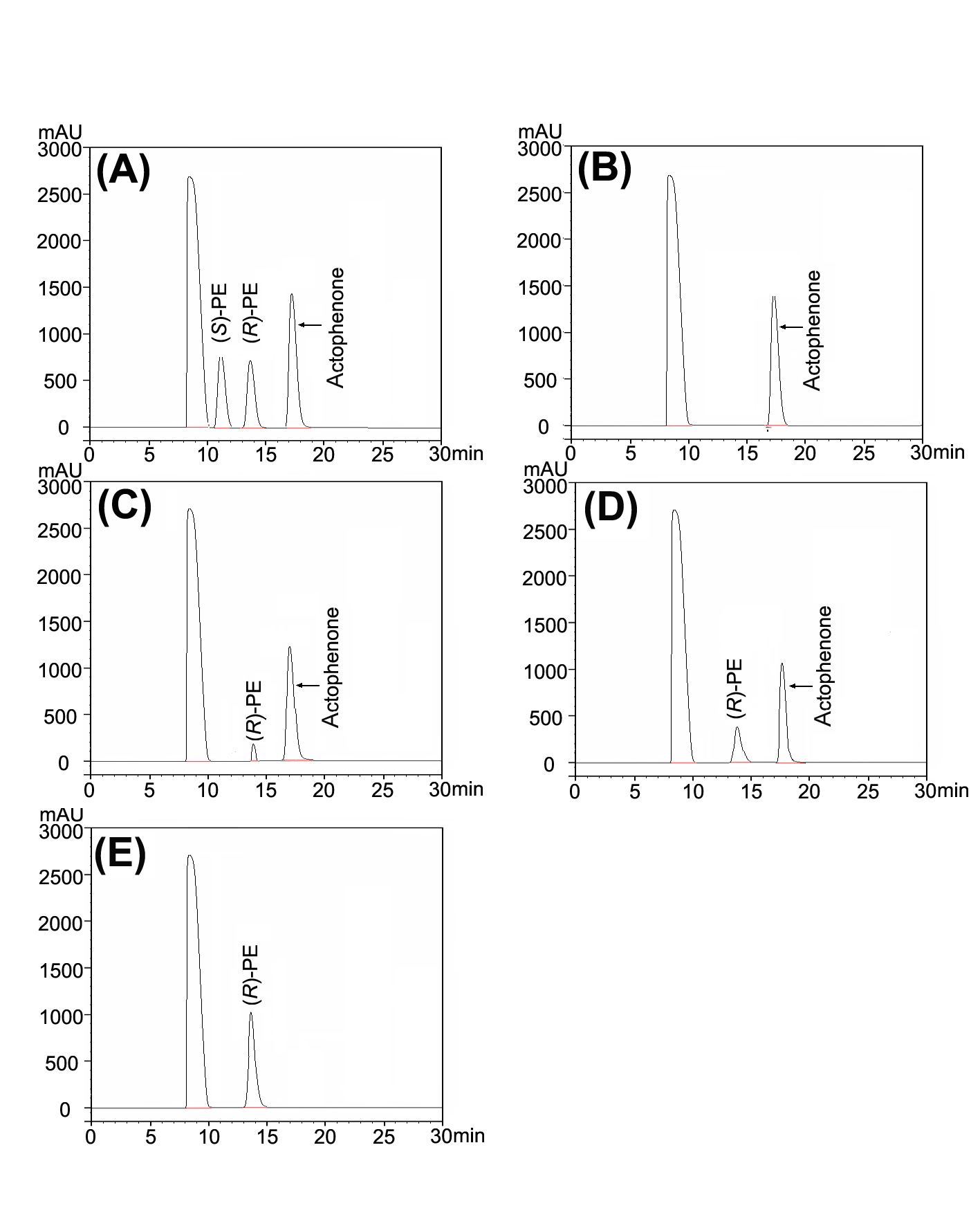


**Table S1.** Plasmids, strains and primers used

| **Plasmids, strains, primers** | **Description** | **Sources** |
| --- | --- | --- |
| **Plasmids** |  |  |
| pET-28a | 5.2 kb, Kam^r^ | Invitrogen |
| pET-SCRⅡ | 6.2 kb, pET28a containing *scr*Ⅱ, Kam^r^ | This work |
| pET-A220D | 6.2 kb, pET28a containing A220D *scr*Ⅱ, Kam^r^ | This work |
| pET-E228S | 6.2 kb, pET28a containing *scr*Ⅱ, Kam^r^ | This work |
| pET-GDH | 6.2 kb, pET28a containing *gdh*, Kam^r^ | This work |
| pET-MS-SD-AS-G | 7.2 kb, pET28a containing E228S *scr*Ⅱ, AD-AS and *gdh* genes, Kam^r^ | This work |
| pET-G-SD-AS-MS | 7.2 kb, pET28a containing *gdh*, AD-AS and E228S *scr*Ⅱ genes,, Kam^r^ | This work |
| pET-MS-L-G | 7.2 kb, pET28a containing E228S *scr*Ⅱ, Linker and *gdh* genes, Kam^r^ | This work |
| pET-G-L-MS | 7.2 kb, pET28a containing *gdh*, Linker and E228S *scr*Ⅱ genes, Kam^r^ | Novagen |
| **Strains** |  |  |
| *Bacillus* sp. YX-1 | DNA donors of *gdh* gene | This laboratory |
| *E. coli /*pETA220D | DNA donors of A220D *scr*Ⅱ gene | This laboratory |
| *E. coli /*pETE228S | DNA donors of E228S *scr*Ⅱ gene | This laboratory |
| *E. coli* BL21(DE3) | F^-^ *ompT hsdS_B_*(*r_B_*^-^ *m_B_*^-^) *gal dcm* (DE3) | Novagen |
| *E. coli* /pET-MS-SD-AS-G | *E*. *coli* BL21 bearing pET-MS-SD-AS-G | This work |
| *E. coli* /pET-G-SD-AS-MS | *E*. *coli* BL21 bearing pET-G-SD-AS-MS | This work |
| *E. coli* /pET-MS-L-G | *E*. *coli* BL21 bearing pET-MS-L-G | This work |
| *E. coli* /pET-G-L-MS | *E*. *coli* BL21 bearing pET-G-L-MS | This work |
| **Primers** | **5’ → 3’** |  |
| MS-L-G_F1 | ATCCTGCTAGCATGGGCGAAATCGAATCTTATTGC (*Nhe*I) | This work |
| MS-L-G_R2 | TGACTCTCGAGACCGCGGCCTGCCTG (*Xho*I) | This work |
| MS-L-G_F2 | **GGCGGTGGTGGCTCTGGCGGTGGTGGCTCTGGCGGTGGTGGCTCT**ATGTATCCGGATTTAAAAGGAAAAGTCGTC | This work |
| MS-L-G_R2 | **AGAGCCACCACCGCCAGAGCCACCACCGCCAGAGCCACCACCGCC**TGGACAAGTGTAACCACCATCGAC | This work |
| G-L-MS_F1 | ATCCTGCTAGCATGTATCCGGATTTAAAAGGAAAAGTCGTC (*Nhe*I) | This work |
| G-L-MS_R1 | TGACTCTCGAGTGGACAAGTGTAACCACCATCGAC (*Xho*I) | This work |
| G-L-MS_F2 | **GGCGGTGGTGGCTCTGGCGGTGGTGGCTCTGGCGGTGGTGGCTCT**ATGGGCGAAATCGAATCTTATTGC | This work |
| GDH-L-SCR_R2 | **AGAGCCACCACCGCCAGAGCCACCACCGCCAGAGCCACCACCGCC**ACCGCGGCCTGCCTG | This work |
| MS-SD-AS-G_F1 | ATCCTGCTAGCATGGGCGAAATCGAATCTTATTGC (*Nhe*I) | This work |
| MS-SD-AS-G_R1 | TGACTCTCGAGACCGCGGCCTGCCTG (*Xho*I) | This work |
| MS-SD-AS-G_F2 | TGTCCATAG**GAAGGAGATATACC**ATGTATCCGGATTTAAAAGGAAAAGTC | This work |
| MS-SD-AS-G_R2 | CGGATACAT**GGTATATCTCCTTC**CTATGGACAAGTGTAACCACCATCGAC | This work |
| G-SD-AS-MS_F1 | ATCCTGCTAGCATGTATCCGGATTTAAAAGGAAAAGTCGTC (*Nhe*I) | This work |
| G-SD-AS-MS_R1 | TGACTCTCGAGTGGACAAGTGTAACCACCATCGAC (*Xho*I) | This work |
| G-SD-AS-MS_F2 | CGCGGTTAA**GAAGGAGATATACC**ATGGGCGAAATCGAATCTTATTGC | This work |
| G-SD-AS-MS_R2 | GCCCAT**GGTATATCTCCTTC**TTAACCGCGGCCTGCCTG | This work |

**Notes：**The sequence of SD-AS or linker is bold; the restriction endonuclease sites are, underlined.

Kam^r^ means kanamycin resistence.
